# Distinct signaling events promote resistance to mitoxantrone and etoposide in pediatric AML: a Children’s Oncology Group report

**DOI:** 10.18632/oncotarget.21363

**Published:** 2017-09-28

**Authors:** Xin Long, Robert B. Gerbing, Todd A. Alonzo, Michele S. Redell

**Affiliations:** ^1^ Department of Pediatrics, Baylor College of Medicine, Houston, TX, USA; ^2^ Texas Children's Hospital, Houston, TX, USA; ^3^ Children's Oncology Group, Monrovia, CA, USA; ^4^ Department of Preventive Medicine, University of Southern California, Los Angeles, CA, USA

**Keywords:** marrow stroma, chemotherapy resistance, ERK1/2, DNA damage, myeloid leukemia

## Abstract

Despite aggressive chemotherapy including mitoxantrone and etoposide, relapse occurs for almost half of children with acute myeloid leukemia (AML). Since both drugs inhibit topoisomerase II and cause DNA double strand breaks, resistance could be achieved by enhanced DNA damage repair (DDR), via homologous recombination (HR) and/or non-homologous end joining (NHEJ). An important source of extrinsic chemoresistance is the bone marrow stroma. We aimed to reveal intrinsic and stroma-induced signaling pathways that contribute to chemoresistance. Sixty diagnostic pediatric AML samples were cultured on or off stromal cells, with or without chemotherapy. We measured apoptosis, DNA damage signaling, and NHEJ/HR pathway activity by FACS analysis of intracellular cleaved PARP, γH2AX, pDNA-PKcs and pATM, respectively. Mitoxantrone strongly increased γH2AX and pDNA-PKcs. Neither chemotherapy drug induced pATM. DNA-PK inhibition alleviated mitoxantrone resistance for AML cells on and off stromal cells. Regarding stroma-induced signaling pathways, ERK1/2 was most consistently activated in primary AML cells by stromal cells. ERK1/2 inactivation partially restored chemosensitivity to AML cells on stromal cells. Additionally, low stroma-induced STAT3 activity and strong stroma-induced mitoxantrone resistance were associated with inferior clinical outcome. Taken together, the NHEJ DDR and ERK1/2 pathways are potential targets for reducing intrinsic and extrinsic chemotherapy resistance in pediatric AML.

## INTRODUCTION

Despite aggressive treatments, recurrence still occurs for almost half of children with AML due to resistance to chemotherapy. Interactions between AML blasts and the bone marrow stromal microenvironment are known to protect blasts from chemotherapy, but the mechanisms remain largely unknown [1, 2]. Mitoxantrone and etoposide are standard chemotherapy agents used in upfront treatment regimens for pediatric AML. Although both are topoisomerase II inhibitors and cause double strand breaks (DSBs) [3], stroma-mediated resistance occurs through different mechanisms. We previously reported that resistance to mitoxantrone is mediated by both stromal soluble factors and cell-cell contact, whereas resistance to etoposide is mediated mainly by physical contact with stroma [4]. Furthermore, we found that stromal CYR61, a small protein in the extracellular matrix that binds integrins, promotes resistance to mitoxantrone, but not etoposide, in pediatric AML [4].

Mammalian cells have two main processes for repairing DSBs. Homologous recombination (HR) is highly accurate but is generally limited to dividing cells because one sister chromatid is used as a template for repair. Nonhomologous end-joining (NHEJ) is more error-prone and occurs in all phases of the cell cycle (reviewed in [5]). The nuclear serine-threonine kinases ataxia-telangiectasia mutated (ATM) and DNA-dependent protein kinase (DNA-PK) mediate proximal activation steps for the HR and NHEJ DSB repair pathways, respectively [6]. Both ATM and the catalytic subunit of DNA-PK (DNA-PKcs) phosphorylate and activate histone H2AX at Ser 139 (γH2AX) [7, 8]. Detection of γH2AX foci by immunofluorescence is highly sensitive and frequently used for measuring DNA damage signaling [9]. Inhibitors of DSB repair pathways are reported to potentiate chemotherapy-induced apoptosis [10–13], supporting the idea that DNA damage repair is a common mechanism of chemotherapy resistance. However, little is known about the importance of DNA damage repair as a mechanism of resistance in pediatric AML. Since HR repair is limited to dividing cells only, we hypothesized that NHEJ repair contributes to chemotherapy resistance in pediatric AML.

While the effects of the microenvironment on DNA damage signaling are not well known, it is well established that stromal cells induce other prosurvival signaling pathways in leukemia cells, through secreted factors and contact-dependent interactions [2, 4]. For example, signal transducer and activator of transcription 3 (STAT3) and STAT5 are activated by a number of secreted factors [14, 15]. Also, extracellular signal-regulated kinases 1/2 (ERK1/2) are important pro-survival protein kinases that are activated by the RAS / RAF / mitogen-activated protein kinase kinase (MAPK/ERK kinase or MEK) pathway downstream of signals from the stroma [16, 17]. The components of the RAS/RAF/MEK/ERK pathway are ubiquitously expressed in mammalian cells. The activation of RAS/RAF/MEK/ERK signaling was observed in about one third of all human cancers [18]. Even in cases without activating mutations of RAS or RAF (e.g., KRAS, NRAS, or BRAF), the RAS/RAF/MEK/ERK pathway is activated in over 50% of AML samples [19]. The aberrant activation of this pathway contributes to cancer as well as other disorders [20]. MEK1/2 are dual-specificity protein kinases [20] and ERK1/2 are their only known downstream targets. ERK1/2 control cell growth [19, 21], cell cycle [22], and have been linked to drug resistance in some cancer models, including docetaxel resistance in breast cancer [23–25]. Therefore, the MEK1/2-ERK1/2 pathway is likely to confer chemoresistance in AML. The contribution of this pathway to stroma-mediated chemoresistance in pediatric AML has not been defined, but is important to determine since inhibitors of the MEK1/2-ERK1/2 signaling pathway are available.

Here, we aimed to identify specific signaling pathways that contribute to chemotherapy resistance in pediatric AML. We identified cell-intrinsic and stroma-induced mechanisms of chemotherapy resistance in a large series of pediatric primary AML samples from patients enrolled on the Children's Oncology Group (COG) AAML03P1, AAML0531 and AAML1031 studies for de novo AML [26, 27]. We demonstrated that chemoresistance via enhanced DNA damage repair is more important for mitoxantrone than etoposide, whereas stroma-induced ERK1/2 activity likely contributes to resistance to both agents. Further, high inducible STAT3 activity and low stroma-induced mitoxantrone resistance correlate with superior clinical outcome, appearing as potential prognostic markers for pediatric AML. Our results suggest NHEJ DNA repair and MEK1/2-ERK1/2 pathways as potential therapeutic targets to overcome chemotherapy resistance and improve clinical outcomes in children with AML.

## RESULTS

### NHEJ repair pathway contributes to intrinsic resistance to mitoxantrone

**Figure 1:**
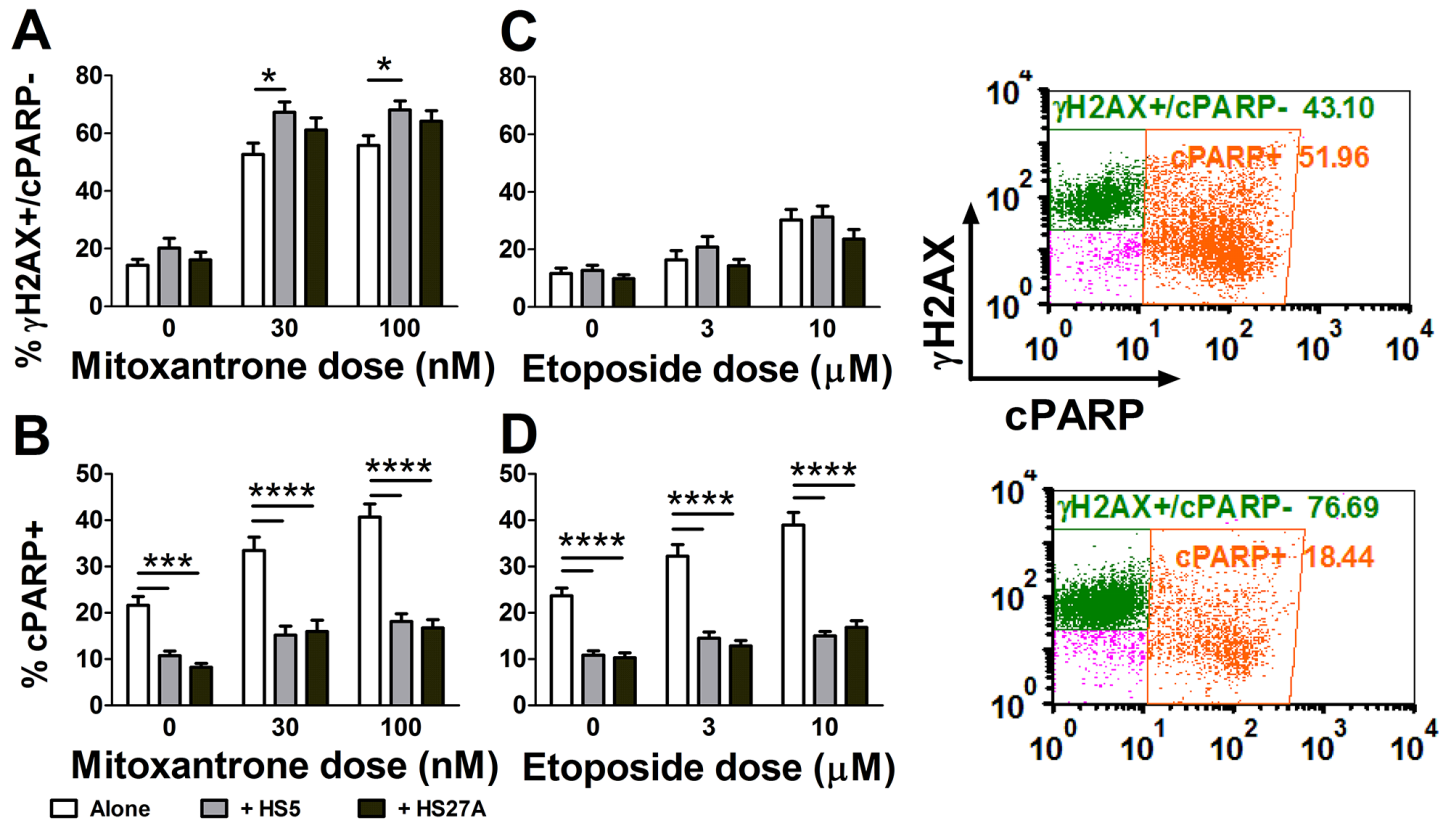
Mitoxantrone robustly activates DNA damage signaling in primary pediatric AML samples Pediatric primary AML cells were cultured alone or co-cultured on HS5 or HS27A stromal cells, then treated with mitoxantrone (n=32) or etoposide (n=33) for 24 hours, before markers for DNA damage signaling (γH2AX) and apoptosis (cPARP) were measured by FACS. The percent of γH2AX+ cells in the live AML cell population (cPARP-) **(A, C)** and the percent of apoptotic cells (cPARP+) in the entire AML cell population **(B, D)** were compared between co-culture vs. alone. Bars show Mean ± SE. Representative dot plots of γH2AX and cPARP are shown for a patient sample cultured off (top) or on (bottom) HS5 cells. ^*^, p<0.05; ^***^, p<0.001; ^****^, p<0.0001.

Since DNA damage repair may contribute to chemotherapy resistance, we compared the DNA damage signaling marker, γH2AX, and the apoptosis marker, cPARP, in primary AML cells on or off stroma, treated with mitoxantrone for either 4 or 24 hours. There was a dose-dependent increase in DNA damage signaling (percent γH2AX+ in live population) at both time points, for AML cells on or off stromal cells (Supplementary Figure 1A-1B, Figure 1A). However, mitoxantrone-induced apoptosis (percent cPARP+) was not apparent at 4 hours, but was significantly increased in AML cells off stroma at 24 hours (Supplementary Figure 1C-1D, Figure 1B). Therefore, we chose the 24-hour time point for the subsequent investigations of chemotherapy resistance.

We next compared chemotherapy-induced apoptosis between mitoxantrone and etoposide with 24-hour treatment. As shown in Figure 1A, mitoxantrone treatment significantly increased the γH2AX+/cPARP- fraction (upper inset), and this was further enhanced by co-culture with HS5 stromal cells (lower inset). In contrast, etoposide treatment only slightly increased γH2AX, and stromal cells did not further increase γH2AX (Figure 1C). The apoptosis rates and the level of protection by stroma were similar for the two chemotherapy agents at these doses (Figure 1B, 1D). For mitoxantrone-treated primary AML cells on stroma, the upregulation of DNA damage signaling was associated with protection from apoptosis (Supplementary Figure 1B, 1D), supporting the hypothesis that DNA damage repair contributes to mitoxantrone resistance.

To determine if stromal co-culture protects AML cells from chemotherapy by inducing a quiescent state, we measured Ki67 as a cell proliferation marker, in 19 primary pediatric samples off and on stroma for 48 hours. Primary AML cells on stroma had a higher percentage of proliferating (Ki67+) cells compared to cells cultured alone (Supplementary Figure 2), suggesting that in our model, AML cells are more proliferative on stroma, rather than more quiescent. Taken together, our results demonstrate that the ability of pediatric AML patient samples to repair mitoxantrone-induced DNA damage, survive, and proliferate, was significantly enhanced by co-culture with stromal cells.

**Figure 2:**
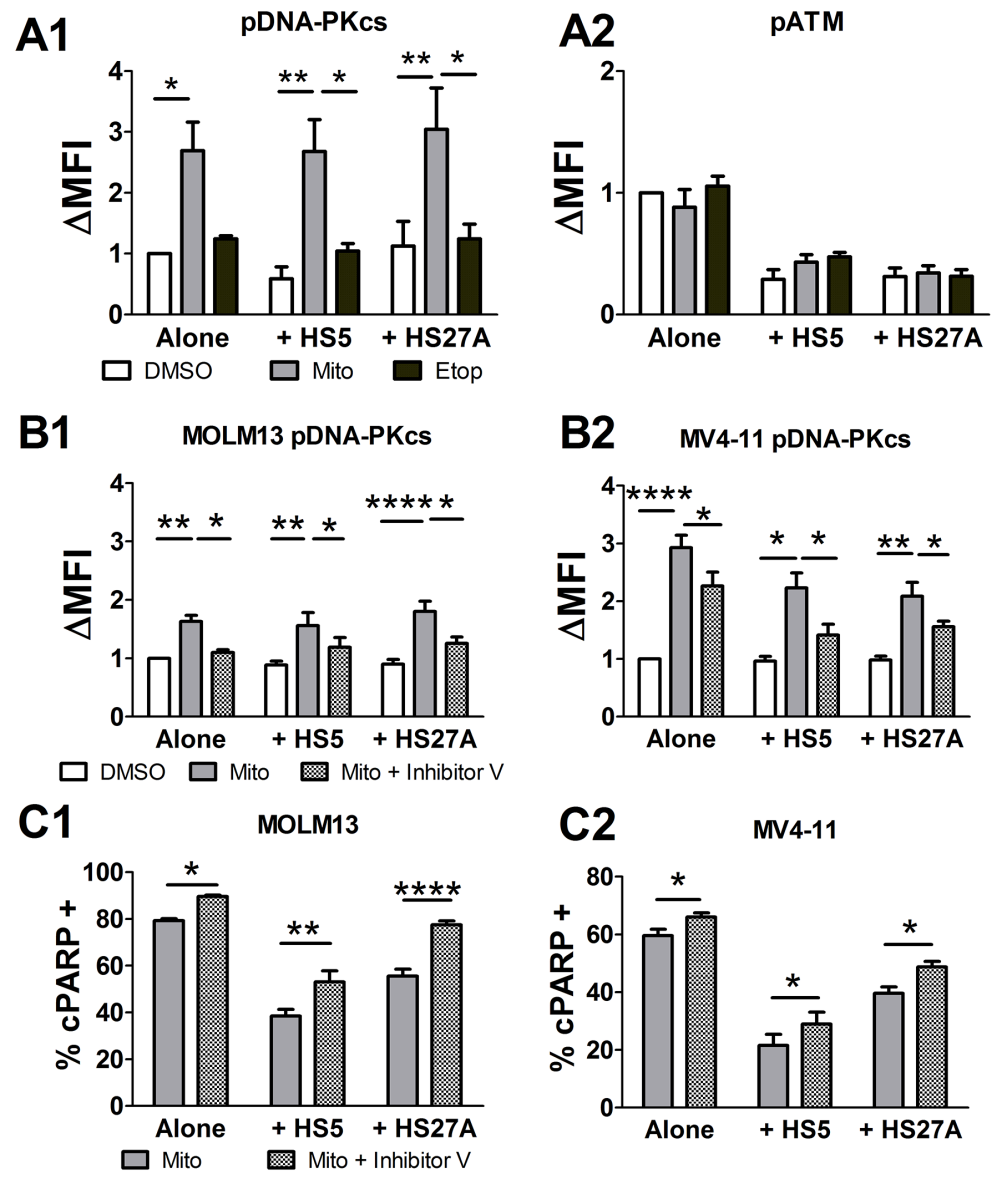
Mitoxantrone-induced DNA-PKcs activation contributes to chemoresistance in pediatric primary AML samples Five pediatric primary AML samples were cultured off or on stroma for 1 hour, followed by chemotherapy treatment (100 nM mitoxantrone, or 10 μM etoposide) for 24 hours, before pDNA-PKcs and pATM were determined in γH2AX+ / cPARP- AML cell population by FACS **(A1-A2)**. Two AML cell lines (MOLM13, MV4-11) were cultured off or on stroma for 24 hours, followed by 10 μM DNA-PK inhibitor V or DMSO and 100 nM mitoxantrone (one hour apart) for 24 hours, before the analysis of pDNA-PKcs **(B1-B2)** in γH2AX+ / cPARP- AML population and of percent cPARP+ cells in whole AML population **(C1-C2)** by FACS. ΔMFI is the fold difference in MFI compared to cells cultured alone and treated with DMSO (A1-A2, B1-B2). For panels C1-2, the percent of spontaneous apoptosis (without chemotherapy) was subtracted from the mitoxantrone-treated samples to yield the percent of apoptosis attributed to mitoxantrone. N=6 for cell lines. Bars show Mean ± SE. ^*^, p<0.05; ^**^, p<0.01; ^****^, p<0.0001.

As both mitoxantrone and etoposide are known to induce DSBs and activate γH2AX, we next looked at the two main DSB repair processes, HR and NHEJ. We first determined the phosphorylation of ATM (HR pathway) and DNA-PKcs (NHEJ pathway) in AML samples on or off stroma and treated with mitoxantrone or etoposide. Mitoxantrone, but not etoposide, induced DNA-PKcs phosphorylation (mean fluorescence intensity, MFI, 25.7 ± 4.2 untreated, v. 61.7 ± 5.1 mitoxantrone, p<0.001, v. 31.5 ± 4.8 etoposide; Figure 2A1). Stromal exposure did not further increase the level of phospho-DNA-PKcs (pDNA-PKcs; Figure 2A1). Therefore, the effect of γH2AX activation by stroma is not due to DNA-PKcs phosphorylation. Neither chemotherapy agent induced phosphorylation of ATM, whether on or off stroma (Figure 2A2). Next, we used a selective inhibitor of DNA-PK, DNA-PK inhibitor V, to assess the role of DNA-PK in mitoxantrone resistance. AML cell lines (MOLM-13 and MV4-11) were cultured off or on stroma and treated with mitoxantrone and DNA-PK inhibitor V or DMSO before the analysis of pDNA-PKcs, γH2AX and cPARP. DNA-PK inhibitor V reduced DNA-PKcs activation in AML cells treated with mitoxantrone and cultured off or on stroma (Figure 2B1-2B2). DNA-PK inhibitor V did not affect γH2AX activation induced by mitoxantrone (Supplementary Figure 3A-3B), indicating that other kinases are involved in that step of DNA damage repair signaling. DNA-PK inhibitor V did not induce apoptosis as a single agent. Importantly, addition of DNA-PK inhibitor V to mitoxantrone significantly increased apoptosis (%cPARP+) and thus alleviated mitoxantrone resistance for AML cells on or off stroma (Figure 2C1-2C2). Similar mitoxantrone sensitizing results were obtained using a different DNA-PK inhibitor, NU7441, in the pediatric AML samples (Supplementary Figure 4). These results demonstrate that the NHEJ repair pathway may represent an intrinsic mechanism of chemoresistance and a potential target for enhancing sensitivity to mitoxantrone in pediatric AML.

### Stroma-induced ERK1/2 activation contributes to chemotherapy resistance

**Figure 3:**
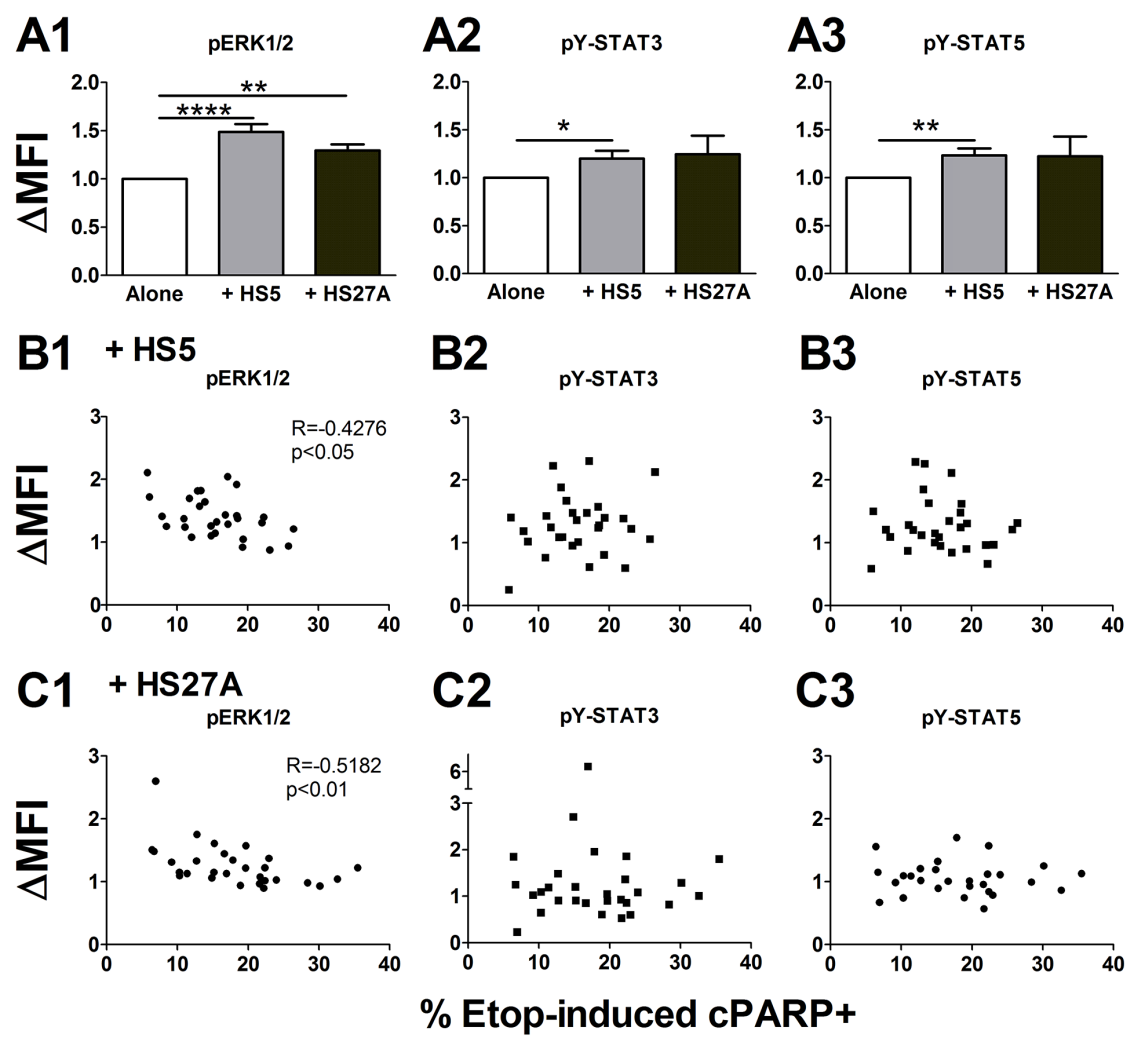
Stroma-induced ERK1/2 activation is inversely proportional to etoposide resistance in pediatric primary AML samples Pediatric primary AML cells were cultured off or on stroma for 24 hours and pERK1/2 **(A1)**, pY-STAT3 **(A2)**, and pY-STAT5 **(A3)** were measured by FACS (n=34). Stroma-induced signaling was expressed as the fold change in MFI over cells cultured alone (ΔMFI). Bars show the mean ± SE. In parallel, cells were cultured off or on stroma for 1 hour and treated with 10 μM etoposide for 24 hours before the analysis of cPARP by FACS. Bivariate correlation analysis was done between the apoptosis rate (%cPARP+) induced by etoposide for AML cells on stroma and the levels of pERK1/2, pY-STAT3, and pY-STAT5 induced by HS5 co-culture **(B1-B3)** or HS27A co-culture **(C1-C3)** (n=29). ^*^, p<0.05; ^**^, p<0.01; ^****^, p<0.0001.

To identify survival pathways that may contribute to extrinsic chemotherapy resistance, we next investigated the activation of key signaling proteins by stroma, including phosphotyrosine-STAT3 (pY-STAT3), pY-STAT5 and pERK1/2. Primary AML cells were cultured on or off stroma for 24 hours before the measurement of these intracellular signaling proteins. ERK1/2 was activated by co-culture with both HS5 and HS27A (Figure 3A1), while STAT3 and STAT5 were activated by HS5 but not HS27A (Figure 3A2, 3A3). HS5 cells secrete dozens of cytokines and growth factors that stimulate these signaling pathways, including interleukin-6 (IL-6) and granulocyte-colony stimulating factor (G-CSF) [4]. In contrast, HS27A cells secrete very few such soluble factors, and conditioned medium from HS27A cells does not activate the STAT3, STAT5 or ERK1/2 pathways (Supplementary Figure 5A-5C). Therefore, these results suggest that contact dependent interactions between AML cells and stromal cells lead to increased ERK1/2 signaling. As our earlier work demonstrated that stroma-dependent etoposide resistance relies on direct cell-cell contact rather than soluble factors [4], we focused on the ERK1/2 pathway as a potential mechanism of etoposide resistance.

We cultured primary AML cells on stromal cells and treated with etoposide for 24 hours, then measured signaling pathway activity and the apoptosis marker cPARP. Bivariate correlation analysis showed that higher levels of stroma-induced pERK1/2 were correlated with lower levels of etoposide-induced apoptosis, i.e., more etoposide resistance (Figure 3B1, 3C1). Neither pY-STAT3 nor pY-STAT5 was correlated with etoposide-induced apoptosis (Figure 3B2, 3B3, 3C2, 3C3). Stroma-induced pERK1/2 was not correlated with stroma-induced pY-STAT3 or pY-STAT5 (Supplementary Figure 6A1-6A2, B1-B2), while stroma-induced pY-STAT3 and pY-STAT5 were correlated with each other (Supplementary Figure 6A3, 6B3). Further, we found strong correlations between pERK1/2, pY-STAT3, pY-STAT5, and γH2AX levels in cells cultured on HS5 and on HS27A (Supplementary Figure 7A-7D). These results suggest that activation of ERK1/2 is likely to promote resistance to etoposide and is functionally unrelated to STAT3 or STAT5 activation. Since both HS5 and HS27A stromal cells activated similar signaling pathways, the signaling responses to co-culture are likely to be induced more by contact than by soluble factors.

**Figure 4:**
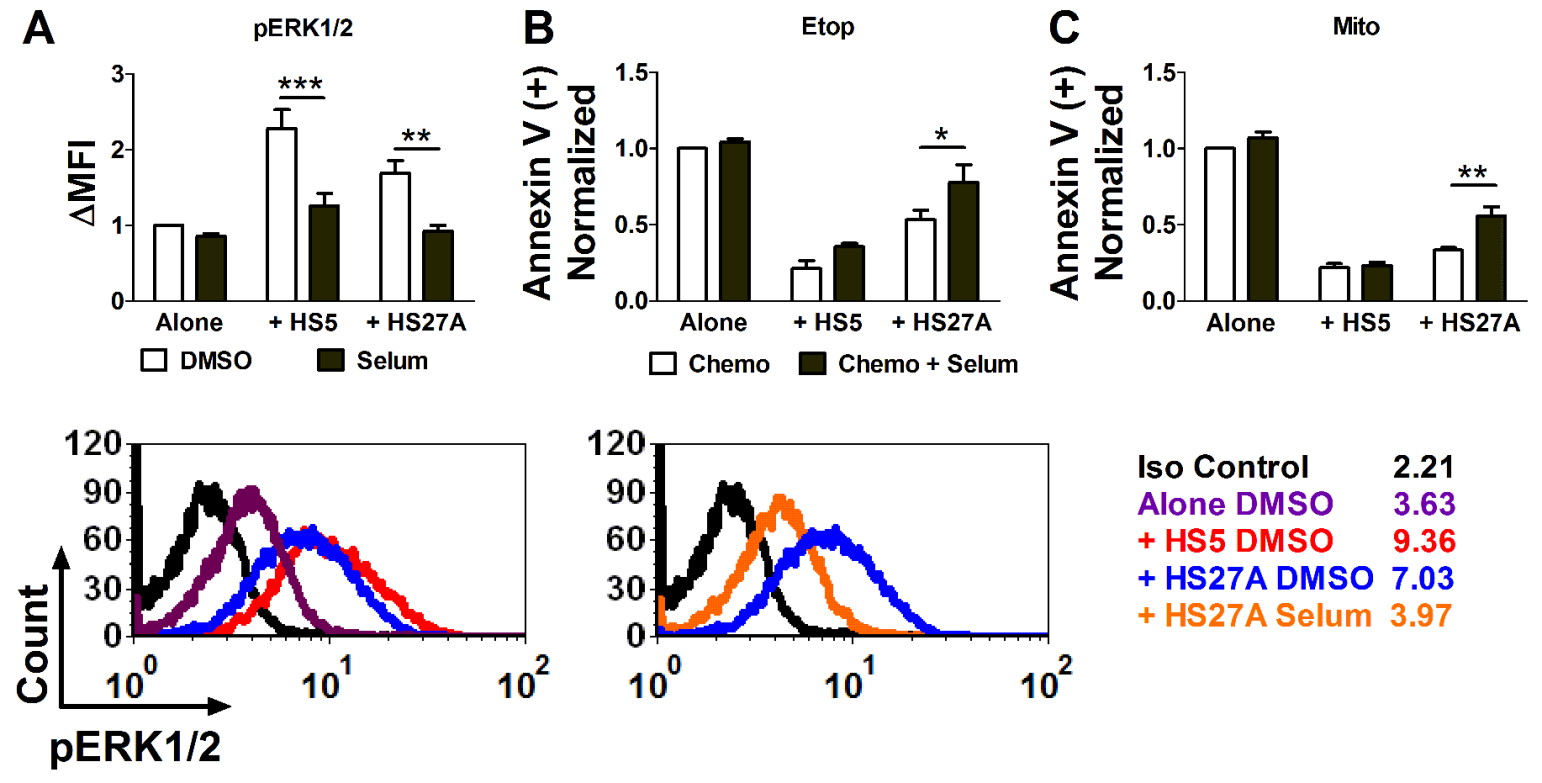
The MEK1/2 inhibitor selumetinib reverses stroma-induced ERK1/2 activation and reduces stroma-mediated chemotherapy resistance in pediatric primary AML samples Pediatric primary AML cells were cultured off or on stroma and treated with 1 μM selumetinib or DMSO for 24 hours, and pERK1/2 was determined by FACS **(A)**. ΔMFI is the fold difference in the MFI of pERK1/2 compared to cells cultured alone and treated with DMSO. The representative histogram overlays (bottom) are depicted for cells on or off stroma (left) and for cells on HS27A stromal cells in the presence or absence of selumetinib (right). The absolute MFI is shown with each line label. In parallel, AML patient samples were cultured off or on stroma for 1 hour and treated with either etoposide (10 μM), mitoxantrone (100 nM), or DMSO, followed by 1 μM selumetinib or DMSO 4 hours later, and apoptosis was measured by Annexin V staining 24 hours after chemotherapy treatment **(B, C)**. The percentage of spontaneous apoptosis (DMSO only) was subtracted from the drug-treated samples to yield the percentage of apoptosis attributed to drug treatment. The Annexin V (+) fold change is the fold difference in chemotherapy-induced apoptosis compared to cells in Alone / Chemotherapy condition. N=4. Bars show Mean ± SE. ^*^, p<0.05; ^**^, p<0.01; ^***^, p<0.001.

As ERK1/2 activation induced by stroma was inversely correlated with etoposide-induced apoptosis, we further determined if ERK1/2 activation may contribute to chemotherapy resistance induced by stroma. Since ERK1/2 enzymes are directly phosphorylated by MEK1/2 [17], we used a selective MEK1/2 inhibitor, selumetinib, for inhibition of ERK1/2 activation. Pediatric primary AML samples were cultured off or on stroma and treated with selumetinib or DMSO before the analysis of pERK1/2. Selumetinib inhibited ERK1/2 activation induced by both HS5 and HS27A cells (Figure 4A and both insets). We further investigated if inhibition of ERK1/2 activation reverses stroma-mediated chemotherapy resistance. Pediatric primary AML cells under similar culture conditions were treated with either etoposide or mitoxantrone and determined for apoptosis by Annexin V staining. Selumetinib did not increase chemotherapy-induced apoptosis in cells off stroma, nor in cells on HS5 stromal cells (Figure 4B, 4C). HS5 stromal cells secrete many soluble factors, which activate multiple pro-survival signaling pathways that contribute to chemoresistance. Therefore, it is likely that blocking only ERK1/2 is not sufficient to overcome stroma-mediated chemoresistance in the HS5 co-culture model. In contrast, selumetinib partially restored sensitivity to AML cells on HS27A stromal cells (Figure 4B, 4C). This result suggests that stroma-induced ERK1/2 signaling contributes to chemotherapy resistance and could be overcome by targeted inhibition.

### Stroma-induced resistance to mitoxantrone, and low stroma-induced STAT3 activity, were associated with worse clinical outcome

**Figure 5:**
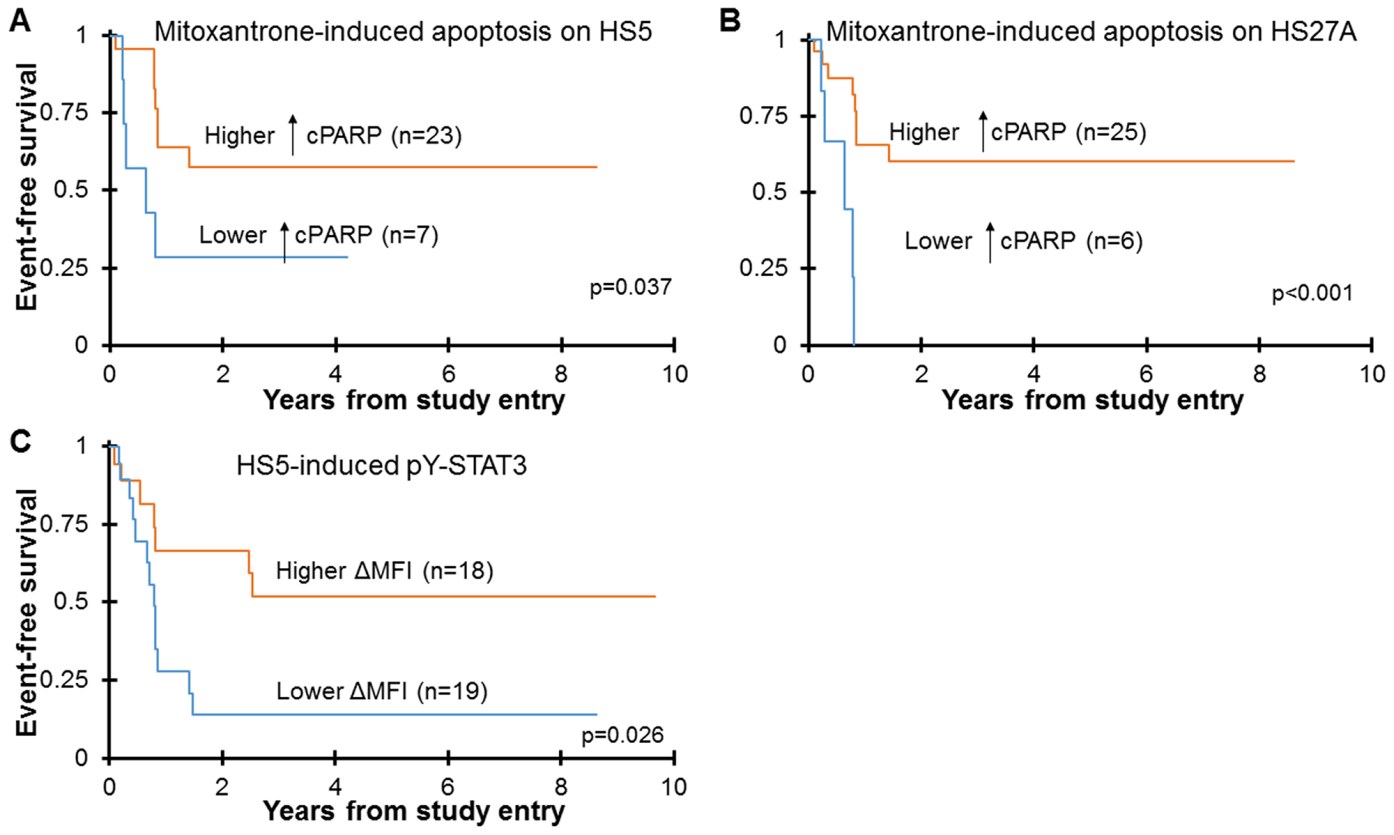
Poor EFS is associated with strong stroma-mediated mitoxantrone resistance and with absent stroma-induced STAT3 activation Pediatric primary AML samples were cultured off or on stroma, treated with 100 nM mitoxantrone for 24 hours, and apoptosis was quantified by FACS analysis for cPARP **(A, B)**. The percentage of spontaneous apoptosis was subtracted from the drug-treated samples to yield the percentage of apoptosis attributed to drug treatment (↑cPARP). Cut point analyses separated the cohort into approximate quartiles. Patients whose AML cells had >3.75% mitoxantrone-induced apoptosis on HS5 stroma had higher 3-year EFS (57.4% ± 24.7%, Mean 3-year EFS ± 2SE), and those with <3.75% mitoxantrone-induced apoptosis on HS5 stroma had lower 3-year EFS (28.6% ± 34.1%) (A). Similarly, patients whose AML cells had >3.43% mitoxantrone-induced apoptosis on HS27A stroma had higher 3-year EFS (60.1% ± 22.3%), and those with <3.43% mitoxantrone-induced apoptosis on HS27A stroma had lower 3-year EFS (0% ± 0%) (B). Patient samples were also cultured on or off HS5 stroma for 24 hours, and pY-STAT3 was measured by FACS **(C)**. Stroma-induced pY-STAT3 is defined as the fold change in MFI over cells cultured alone (ΔMFI). Cut point analysis separated the cohort in half. The patients whose AML cells failed to induce pY-STAT3 (stroma-induced pY-STAT3 < 1.22) when co-cultured with HS5 cells had significantly lower 3-year EFS (13.9% ± 18.2%) than those whose AML cells appropriately induced pY-STAT3 (> 1.22, 51.9% ± 26.7%). EFS was estimated by the Kaplan-Meier method and groups were compared for significant differences by the log rank test. Patients who underwent SCT on protocol therapy were censored for Kaplan-Meier analysis of EFS.

Since chemotherapy resistance contributes to induction failure, relapse, and death in AML, we aimed to determine if the degree of chemoresistance *in vitro* may be associated with clinical outcome. Basic clinical data associated with the samples used in this study are provided in Supplementary Table 1. We used cut point analyses to determine a level of chemotherapy-induced apoptosis that was significantly associated with outcome. In all outcome analyses, patients who received stem cell transplant (SCT) on protocol therapy were censored. As shown in Figure 5A-5B, patients with the lower levels of mitoxantrone-induced apoptosis on stroma *in vitro* had significantly worse event-free survival (EFS) compared to patients with more mitoxantrone-induced apoptosis. The difference in overall survival was not significant. For the HS27A co-culture model, patients whose blasts had <3.43% increase in cPARP+ cells with mitoxantrone treatment had a 3-year EFS of 0% ± 0%, while those whose blasts had >3.43% increase in cPARP+ cells had a 3-year EFS of 60.1% ± 22.3% (n=6 and 25, respectively; p<0.001). The results with the HS5 co-culture model were similar. Importantly, this relationship was true for samples co-cultured with stromal cells, but for samples cultured alone, the level of mitoxantrone-induced apoptosis was not at all associated with EFS. This suggests that the strength of stroma-induced mitoxantrone resistance *in vitro* is a clinically relevant measurement that may predict patient outcome. Of note, the levels of etoposide-induced apoptosis, on or off stroma, were not significantly associated with clinical outcome (data not shown). Therefore, although both mitoxantrone and etoposide are topoisomerase II inhibitors, resistance occurs through distinct mechanisms, and the *in vitro* assays to evaluate each drug differ in their relationship with clinical outcomes.

Since signaling events induced by stroma may contribute to stroma-mediated chemotherapy resistance and thus correlate with clinical outcome, we next looked at the relationship between stroma-induced signaling events (pY-STAT3, pY-STAT5 and pERK1/2) and EFS. For HS5-induced pY-STAT3, we found that high levels of inducible signaling were significantly associated with better EFS (Figure 5C). Specifically, patients whose blasts demonstrated increased pY-STAT3 (ΔMFI >1.22) had a 3-year EFS of 51.9% ± 26.7% (n=18), while patients whose blasts demonstrated no change or a decrease in pY-STAT3 (ΔMFI < 1.22) with HS5 co-culture had a 3-year EFS of 13.9% ± 18.2% (n=19; p=0.026). This result is consistent with our previous report demonstrating that robust activation of pY-STAT3 in response to both G-CSF and IL-6 stimulation was associated with excellent EFS and overall survival [14]. In the current study, there was no relationship between outcome and HS27A-induced pY-STAT3, implicating soluble factor-induced STAT3 signaling as the prognostically relevant mechanism. Further, stroma-induced pY-STAT5 was not associated with outcome. There was a trend toward an association between high HS27A-induced pERK1/2 and inferior outcome, but it did not reach statistical significance, likely due to the small sample size (data not shown). Therefore, in this study, high soluble factor-induced STAT3 activity again emerged as a favorable prognostic factor for pediatric AML.

To determine whether aberrant signaling related to the FLT3/ITD mutation could be influencing our results, we performed separate Kaplan-Meier EFS analyses for FLT3/ITD-positive and FLT3/ITD-negative patients. As shown in Supplementary Figure 8A1-8A3, all FLT3/ITD-positive patients had poor EFS, regardless of mitoxantrone-induced apoptosis on stroma or HS5-induced pY-STAT3. For the FLT3/ITD-negative population (Supplementary Figure 8B1-8B3), the outcome analyses were similar to our previous findings for the entire cohort (Figure 5). These results indicate that FLT3/ITD mutation did not contribute to the worse prognosis associated with lower mitoxantrone-induced apoptosis on stroma nor lower HS5-induced pY-STAT3.

We performed univariable Cox analyses of EFS, for the response variables measured here and for risk group as defined by molecular features at diagnosis. Specifically, patients with monosomy 7, -5/5q, and/or FLT3/ITD with allelic ratio (AR) > 0.4 were considered high risk; and patients with inv(16), t(8;21), and NPM1 or CEBPα mutations were considered low risk. Otherwise, patients with known cytogenetics without these features were classified as standard risk. A low rate of mitoxantrone-induced apoptosis on HS5 / HS27A, and lower HS5-induced pY-STAT3 were associated with significantly worse EFS (Supplementary Table 2). Molecularly defined risk group was not a significant factor for EFS, likely due to the small number of cases in each group. Next, we performed a multivariable Cox analysis of EFS to adjust for risk group (Supplementary Table 3). Even after accounting for molecular risk features, mitoxantrone-induced apoptosis on HS27A continued to be significantly associated with EFS (hazard ratio 7.62 for lower cPARP group, p=0.005.). While the statistical power provided by these small sample sizes is limited, the data suggest that environment-mediated mitoxantrone resistance is likely to be an important factor in determining a patient's response to treatment.

## DISCUSSION

In the present study, we investigated mechanisms underlying mitoxantrone and etoposide resistance in a large series of pediatric primary AML samples. We identified a cell-intrinsic mechanism of resistance to mitoxantrone, namely NHEJ-mediated DNA damage repair. We also identified an extrinsic mechanism of resistance to both topoisomerase II inhibitors, stroma-induced MEK1/2-ERK1/2 signaling. These results demonstrate that chemotherapy resistance is a complex problem requiring different approaches in different situations. Importantly, both mechanisms described in this study are potentially targetable by small-molecule inhibitors.

An important finding of this study is the difference in DNA damage responses induced by mitoxantrone compared to etoposide. Mitoxantrone induced a much stronger γH2AX response compared to etoposide. A similar difference in γH2AX induction in fibroblasts was attributed to mitoxantrone's ability to damage DNA through a variety of mechanisms [28]. For example, mitoxantrone intercalates into DNA, similar to anthracyclines. Additionally, mitoxantrone [29], but not etoposide [30], induces DNA damage via generation of reactive oxygen species. Taken together, these differences in activity may explain why γH2AX was detected modestly in live cells with etoposide treatment, and robustly with mitoxantrone treatment. In addition, co-culturing AML cells with stromal cells further upregulated γH2AX and reduced apoptosis induced by mitoxantrone. As a DNA damage signaling molecule, γH2AX promotes the recruitment of DNA damage repair complexes to sites of DSBs, thereby initiating DNA damage repair [31] and reducing apoptosis. Similarly, an association between increased γH2AX and decreased apoptosis was shown for melanoma cells treated with arsenite [32].

In order to understand and thus overcome mitoxantrone resistance due to enhanced DNA damage signaling, we investigated whether the response involves the HR pathway or the NHEJ pathway. Our results clearly indicate that, in this cohort of pediatric primary AML samples, the NHEJ repair pathway was activated by mitoxantrone. Our finding that mitoxantrone-induced DNA-PKcs activation was not further enhanced by stromal co-culture suggests that mitoxantrone resistance via NHEJ is cell intrinsic, i.e. largely independent of the influence of stromal cells. This idea is supported by the ability of DNA-PK inhibitor V to sensitize AML cells to mitoxantrone both off and on stroma. In a study of adult AML samples, the authors reported heterogeneity in the preferred DNA repair pathways in response to etoposide [33]. That is, in some cases both HR and NHEJ pathways were inactive, in others both were highly active, and in another subset only NHEJ activity was found. While we did not see evidence of NHEJ activation in response to etoposide in these pediatric cases, our results with mitoxantrone lend additional support to the conclusion that the NHEJ DNA damage repair pathway may be a more relevant target than the HR pathway, for focusing efforts on developing novel strategies to improve the efficacy of chemotherapy for AML.

While DNA damage signaling was largely unaffected by the stromal microenvironment in these experiments, we did find an important relationship between stroma-mediated mitoxantrone resistance and clinical outcome. Specifically, mitoxantrone-induced apoptosis in AML samples co-cultured with stromal cells significantly and positively correlated with EFS. Patients whose samples showed higher rates of mitoxantrone-induced apoptosis on stroma had better EFS than patients with low rates of apoptosis, i.e. more stroma-induced resistance. Similarly, stroma-mediated cytarabine resistance *in vitro* was associated with poor prognosis in adult AML patients [34]. We acknowledge that our statistical analysis is limited by the small sample size, and a larger replication cohort is warranted. Nevertheless, our findings suggest that the strength of stroma-induced chemotherapy resistance *in vitro* may be a clinically relevant measurement reflecting chemotherapy resistance in patients. Therefore, our studies of stroma-dependent chemotherapy resistance, particularly resistance to mitoxantrone, can be leveraged to develop treatment strategies to overcome this resistance.

With regard to mechanisms of stroma-mediated resistance, we demonstrated that key pro-survival signaling pathways, including ERK1/2, STAT3 and STAT5, are activated in AML cells by stromal co-culture. ERK1/2 and STAT3 pathways have been associated with stroma-induced resistance to other drugs, such as FLT3 inhibitors [35, 36]. In our study, increased HS5-induced STAT3 activation was associated with a more favorable clinical outcome. HS5 cells secrete dozens of growth factors and cytokines, whereas HS27A stromal cells secrete very few [4]. Therefore, this result confirms our previous report that patients whose AML cells demonstrated robust STAT3 activation in response to G-CSF and IL-6 stimulation had a significantly better outcome than patients whose blasts did not respond to these stimuli [14]. The mechanism of this relationship is under investigation. Others have reported that relatively weak STAT3 activation may antagonize apoptosis while stronger activation may prevent cell growth [37]. In the meantime, the association between robust STAT3 activation and favorable outcome has been demonstrated in two pediatric cohorts representing different treatment eras (CCG2961 treatment v. COG AAML0531-like treatment), and with two stimuli (individual soluble factors v. stromal co-culture). Therefore, the use of induced STAT3 activity as a prognostic marker for pediatric AML warrants further study.

While this result does not preclude a role for STAT3 signaling in therapy resistance in pediatric AML, it prompted us to look at other pathways. The ERK1/2 pathway was most consistently activated by stromal co-culture in our samples, and our results point to contact interactions as the primary stimulus. ERK1/2 are the only known substrates of MEK1/2 [17], and MEK1/2 inhibition by selumetinib reduced ERK1/2 activity to basal levels, confirming that MEK1/2 is a main activator of ERK1/2, and MEK1/2 inhibition is an effective means of blocking MEK1/2-ERK1/2 pathway in AML-stromal cell co-cultures. Selumetinib alleviated HS27A-induced resistance to both mitoxantrone and etoposide, suggesting that MEK1/2-ERK1/2 activation contributes to stroma-mediated chemotherapy resistance in that model. In contrast, selumetininb did not alleviate chemoresistance for cells on HS5. It is possible that the soluble factors provided by HS5 cells activate multiple pro-survival signaling pathways, such that inhibiting one pathway is not sufficient to significantly affect apoptosis in HS5 co-cultures.

In sum, our results suggest that incorporating small-molecule inhibitors of DNA-PK and MEK1/2-ERK1/2 pathways may alleviate intrinsic and extrinsic resistance to chemotherapy agents, respectively, thereby enhancing the efficacy of chemotherapy regimens and improving clinical outcomes for pediatric AML patients. Importantly, as selumetinib is already in clinical trials for other pediatric malignancies without showing much toxicity [38], our research may lead to improvements in pediatric AML therapy and outcomes relatively quickly.

## MATERIALS AND METHODS

### Reagents

Etoposide, mitoxantrone, and the selective DNA-PK inhibitor V were obtained from Sigma-Aldrich (St. Louis, MO, USA). The selective MEK1/2 inhibitor, selumetinib, and the selective DNA-PK inhibitor, NU7441, were purchased from Selleck (Houston, TX, USA). Dimethyl sulfoxide (DMSO) was purchased from American Type Culture Collection (ATCC, Manassas, VA, USA).

### Cell lines

HS5 and HS27A human bone marrow stromal cells were purchased from ATCC and cultured as previously described [4]. HS5 and HS27A cells were stably transduced with pCDH.CMV.mOrange (gift of Dr. Stephen Gottschalk, Baylor College of Medicine), to allow distinction from AML cells by flow cytometry. For all experiments, HS5-mOrange and HS27A-mOrange cells are denoted as HS5 and HS27A cells. MOLM13 and MV4-11 AML cell lines were purchased from ATCC and cultured in RPMI 1640 (ATCC) with 10% fetal bovine serum (FBS, Invitrogen/Life Technologies, Carlsbad, CA, USA), 2 mM L-glutamine (Invitrogen), 100 units/ml penicillin and 100 μg/ml streptomycin (Invitrogen).

### Primary AML samples

Fifty-eight de-identified pediatric primary AML samples were obtained from the COG AML Reference Lab and two from Texas Children's Hospital. Written informed consent was obtained in accordance with the Declaration of Helsinki, for bone marrow and peripheral blood cells to be stored for research. All patients were treated with the AAML03P1, AAML0531, or AAML1031 protocols, which used the same chemotherapy backbone. All samples were enriched for mononuclear cells by density centrifugation, and cryporeserved. Samples were carefully thawed into warm Iscove's MEM (Hyclone/GE Healthcare Bio-Sciences, Pittsburgh, PA, USA) with 20% FBS (Invitrogen/Life Technologies), and rested for 1 hour before counting viable cells. Only samples with ≥75% viability at thawing and with <40% spontaneous apoptosis (off stroma) after overnight culture were included in the data analysis. These studies were approved by the Institutional Review Board of Baylor College of Medicine.

### Co-culture experiments

For co-culture experiments, primary AML patient samples were co-cultured with HS5 or HS27A cells for 1 hour, before treatment with etoposide (10 μM) or mitoxantrone (100 nM) for 24 hours except as indicated below, as described [4]. In parallel, AML cells were cultured alone and treated with chemotherapy drugs with the same doses and duration. For 18 patient samples with sufficient viable cells, they were treated with mitoxantrone for either 4 or 24 hours before harvesting for the analysis of γH2AX and cPARP by FACS. For experiments using MOLM13 and MV4-11 AML cell lines, cells were co-cultured with stroma for 24 hours before mitoxantrone treatment. DNA-PK inhibitor V was added 1 hour before mitoxantrone treatment (24 hours) for the analysis of γH2AX, cPARP, and DNA-PKcs. Six pediatric AML patient samples were treated with DNA-PK inhibitor NU7441 (1 μM) or DMSO and mitoxantrone before the analysis of cPARP by flow cytometry. For Ki67 assay, 19 patient samples were cultured off or on stroma for 48 hours before harvesting and staining for flow cytometry analysis. The MEK1/2 inhibitor selumetinib was added 4 hours after the chemotherapy agent. The doses for DNA-PK inhibitor V and selumetinib were 10 μM and 1 μM, respectively. DMSO was used as vehicle control. After 24 hours of chemotherapy treatment, AML cells were harvested and prepared for flow cytometry analysis of intracellular markers as described below.

### Annexin V apoptosis assay

For the apoptosis assay, cells were labeled using Annexin V-FITC (BD Biosciences, San Jose, CA, USA). The mOrange-positive stromal cells were excluded, and the percentage of apoptotic AML cells (mOrange-negative, FITC-positive) was determined as described [4]. Data were acquired on the LSRII (BD) and analyzed with Diva (BD Biosciences). The percentage of spontaneous apoptosis was subtracted from the drug-treated samples to yield the percentage of apoptosis attributed to drug treatment, as described [15].

### Analysis of intracellular signaling proteins and Ki67 by flow cytometry

Primary AML patient samples were harvested, fixed, permeabilized, and stained for intracellular proteins as described [14, 15]. For most experiments, cells were stained with anti-CD45 (APC-H7) to exclude lymphocytes (SSC-low, CD45-high), and anti-cleaved PARP (cPARP, Alexa Fluor^®^ 647) to quantify or exclude apoptotic cells. For stroma-induced signaling experiments, cells were stained with anti-pY705-STAT3 (PE-CF594), anti-pY694-STAT5 (PerCP-Cy5.5), and anti-pERK1/2 (Pacific Blue, Cell Signaling Technology, Danvers, MA, USA). For analysis of DNA damage repair related signaling (pDNA-PKcs and pATM), cells were stained with anti-CD45 (PE-CF594) to exclude lymphocytes, then with anti-γH2AX (pS139, Alexa Fluor^®^ 488) and anti-cPARP (Alexa Fluor^®^ 647) to identify the CD45^low^/γH2AX+/cPARP- AML population. The pDNA-PKcs and pATM kinases were stained with rabbit anti-pS2056-DNA-PKcs (Abcam, Cambridge, MA, USA) + goat-anti-rabbit Alexa Fluor^®^ 750 secondary antibody (ThermoFisher Scientific, Waltham, MA, USA), and mouse anti-pS1981-ATM (Rockland Immunochemicals Inc., Limerick, PA, USA) + goat-anti-mouse Alexa Fluor^®^ 405 secondary antibody (ThermoFisher Scientific), respectively. The control samples were stained with isotype control primary and fluorochrome-conjugated secondary antibodies. For Ki67 analysis, fixed and permeabilized samples were stained with anti-Ki67 antibody (Alexa Fluor^®^ 700) and quantified for percent Ki67 positive population. All fluorochrome-conjugated primary antibodies were obtained from BD Biosciences, except those indicated otherwise. Data were acquired on the LSRII. The mOrange-positive stromal cells were excluded, and 10,000 mOrange-negative events were acquired for AML cells. Data were further analyzed with FCS Express 5 (De Novo Software, Glendale, CA, USA).

### Statistical analysis

Values are means ± standard error of the mean (SE). Two-way analysis of variance (ANOVA) was used to compare co-culture v. alone, or selumetinib / DNA-PK inhibitor V v. DMSO under different culture conditions, followed by Bonferroni's least significant difference post hoc test. Wilcoxon matched-pairs signed rank test was done to compare co-culture v. alone for Ki67 analysis. Bivariate correlation was done by Spearman correlation analysis. Analyses were done using GraphPad Prism 5.02 software (GraphPad Software, La Jolla, CA, USA); p < 0.05 was considered statistically significant.

For survival analyses, data were current as of December 31, 2013 for patients enrolled on AAML03P1 and as of December 31, 2016 for patients enrolled on AAML0531 and AAML1031. One of the two local samples was provided by a patient who was not enrolled on any COG study; this patient was excluded from all outcome analyses. The Kaplan-Meier method was used to estimate EFS. EFS was defined as time from study entry until induction failure, relapse, or death. Patients who received SCT on protocol therapy were censored at the time of transplant. Otherwise, patients were censored at last contact. Differences between groups of patients were tested using the log-rank test. Cox proportional hazard models were used to estimate hazard ratios for univariable and multivariable analyses of EFS. These analyses were performed using the SAS statistical program (SAS-PC, version 9.4; SAS Institute, Inc., Cary, NC, USA).

## SUPPLEMENTARY MATERIALS FIGURES AND TABLES





## Abbreviations

AML: acute myeloid leukemia
APC: allophycocyanin
ATCC: American Type Culture Collection
ATM: ataxia-telangiectasia mutated
CI: confidence interval
COG: the Children's Oncology Group
cPARP: cleaved PARP
DDR: DNA damage repair
DMSO: dimethyl sulfoxide
DNA-PKcs: the catalytic subunit of DNA-dependent protein kinase
DSBs: double strand breaks
EFS: event-free survival
ERK1/2: extracellular signal-regulated kinases 1/2
FITC: fluorescein isothiocyanate
FLT3: FMS-like tyrosine kinase-3
G-CSF: granulocyte-colony stimulating factor
γH2AX: histone H2AX phosphorylated at Ser 139
HR: hazard ratio
HR: homologous recombination
IL-6: interleukin-6
MEK1/2: mitogen-activated protein kinase kinase (MAPK/ERK kinase)
NHEJ: non-homologous end joining
PE: phycoerythrin
PerCP: peridinin chlorophyll
PI3K: phosphatidylinositide 3-kinases
pY-STAT3: phosphotyrosine-STAT3
SCT: stem cell transplant
SSC: side scatter
STAT3: signal transducer and activator of transcription 3
