# Supplementary material for: Distinct signaling events promote resistance to mitoxantrone and etoposide in pediatric AML: a Children’s Oncology Group report
Source: Oncotarget. 2017 Sep 28;8(52):90037–49. doi: 10.18632/oncotarget.21363 (PMC5685730; doi:10.18632/oncotarget.21363)
Supplement: Supplementary file 1 [file oncotarget-08-90037-s001.pdf]

Distinct signaling events promote resistance to mitoxantrone and etoposide in pediatric AML: a children's oncology group report

SUPPLEMENTARY MATERIALS

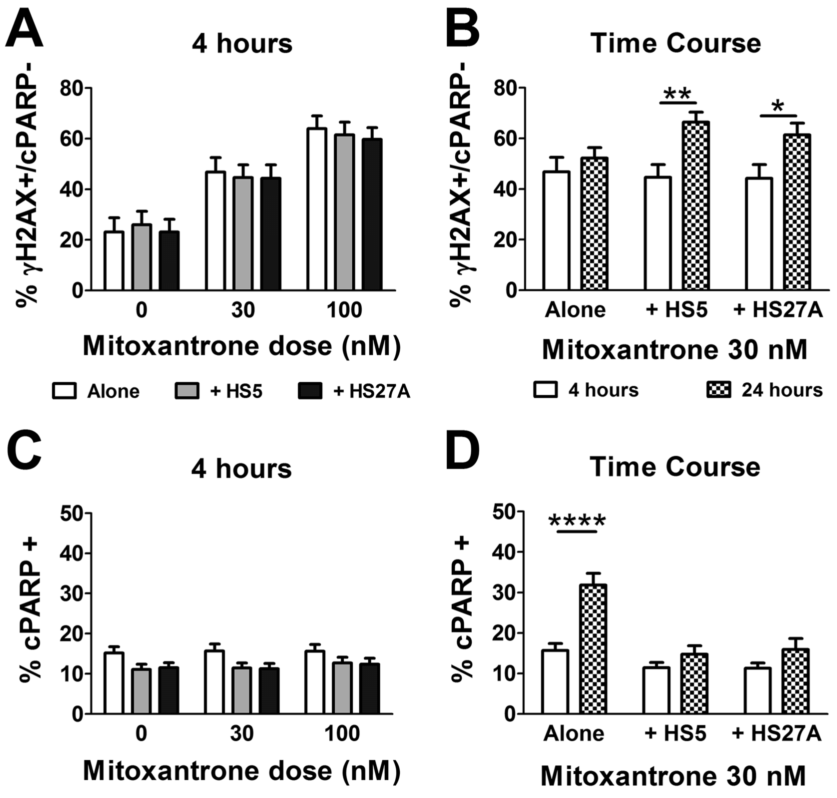

Supplementary Figure 1: Longer mitoxantrone treatment duration resulted in more stroma-induced γH2AX activation and more apoptosis for cells cultured alone. Pediatric primary AML samples were cultured off or on stroma for 1 hour before mitoxantrone treatment for 4 or 24 hours, followed by γH2AX **(A, B)** and cPARP **(C, D)** analysis by FACS. N=18. Bars show Mean ± SE. *, $p<0.05$; **, $p<0.01$; ****, $p<0.0001$.

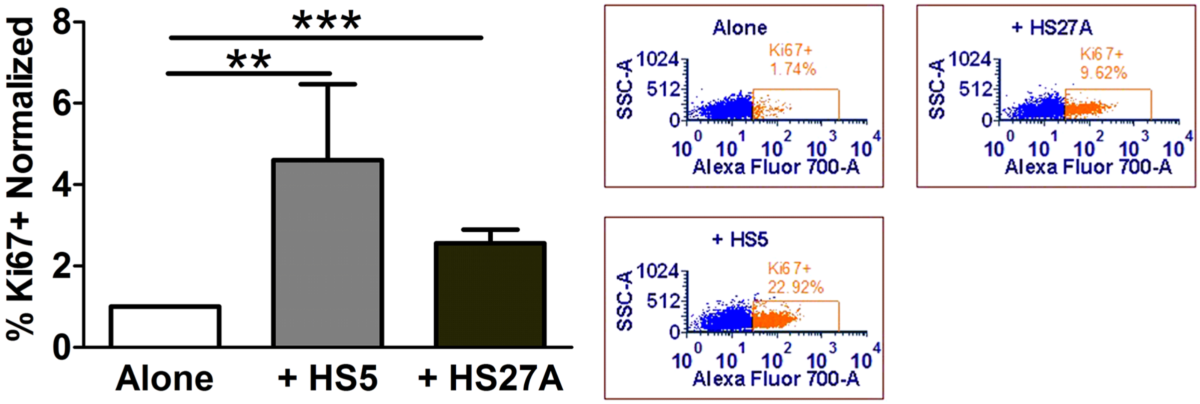

Supplementary Figure 2: Stromal co-culture induced more Ki67 for patient samples. Pediatric primary AML samples were cultured off or on stroma for 48 hours and Ki67 was measured by FACS. N=19. Bars show Mean ± SE. *, $p<0.05$.

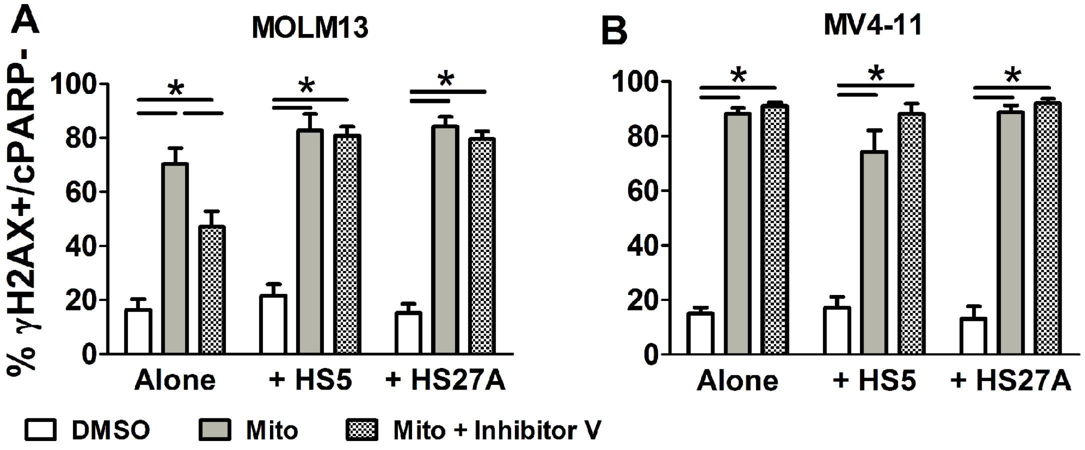

Supplementary Figure 3: DNA-PK inhibition did not change γH2AX in AML cell lines. Two AML cell lines (MOLM13, MV4-11) were cultured off or on stroma for 24 hours, followed by 10 µM DNA-PK inhibitor V or DMSO and 100 nM mitoxantrone (an hour apart) for 24 hours, before the analysis of percent γH2AX+ cells in live (cPARP-) AML population **(A, B)**. N=6. Bars show Mean ± SE. *, $p<0.05$.

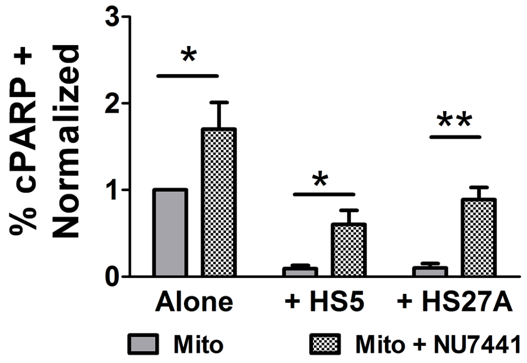

Supplementary Figure 4: DNA-PK inhibitor, NU7441, alleviated mitoxantrone resistance in pediatric primary AML samples. Six pediatric primary AML samples were cultured off or on stroma for 1 hour and treated with 100 nM mitoxantrone and 1 µM NU7441 or DMSO for 24 hours before the analysis of cPARP in all AML cells by FACS. The percent of spontaneous apoptosis (without chemotherapy) was subtracted from the drug-treated samples to yield the percent of apoptosis attributed to drug treatment. The mitoxantrone-induced apoptosis (%cPARP+) was normalized to the apoptosis rate for the Alone / Mito condition, yielding fold change difference. Bars show Mean ± SE. *, $p<0.05$; **, $p<0.01$.

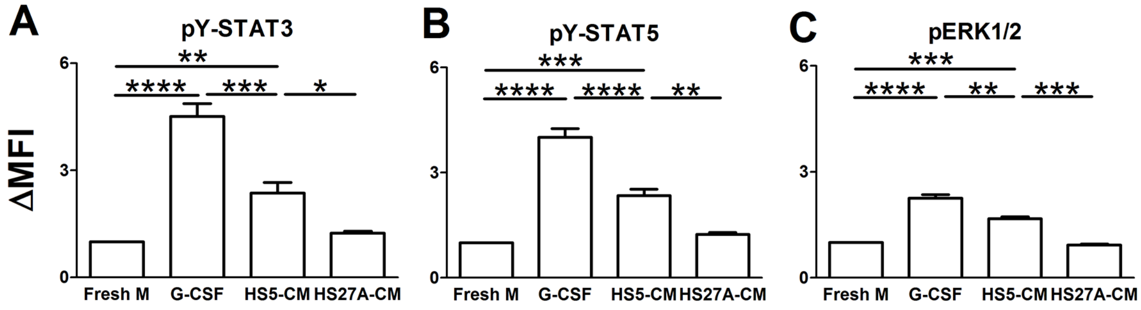

Supplementary Figure 5: HS5-conditioned medium, not HS27A-conditioned medium, activates STAT3, STAT5, and ERK1/2 in Kasumi-1 AML cells. Kasumi-1 AML cell line was treated with fresh medium, HS5-conditioned medium, HS27A-conditioned medium, or G-CSF (100 ng/mL; positive control) for 15 minutes before the analysis of pY-STAT3, pY-STAT5, and pERK1/2 by FACS **(A-C)**. ΔMFI is defined as the fold change in MFI over cells treated with fresh medium. Bars show Mean ± SE. N=4. *, $p<0.05$; **, $p<0.01$; ***, $p<0.001$; ****, $p<0.0001$.

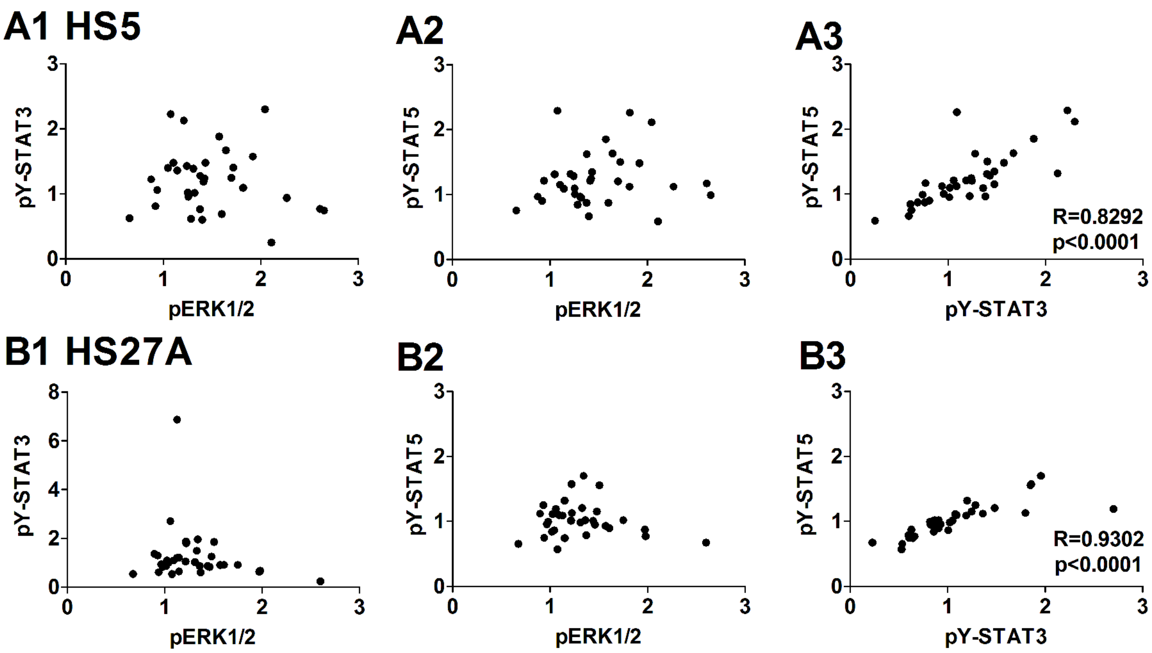

Supplementary Figure 6: Stroma-induced pY-STAT3 and pY-STAT5 are highly correlated with each other but not with stroma-induced pERK1/2. Pediatric primary AML samples were cultured off or on stroma for 24 hr and pY-STAT3, pY-STAT5 and pERK1/2 were measured by FACS. Bivariate correlation analysis was done between the ΔMFI of pERK1/2, pY-STAT3 or pY-STAT5 induced by either HS5 **(A1-A3)** or HS27A cells **(B1-B3)**. ΔMFI is expressed as the fold change in MFI over cells cultured alone. N=34.

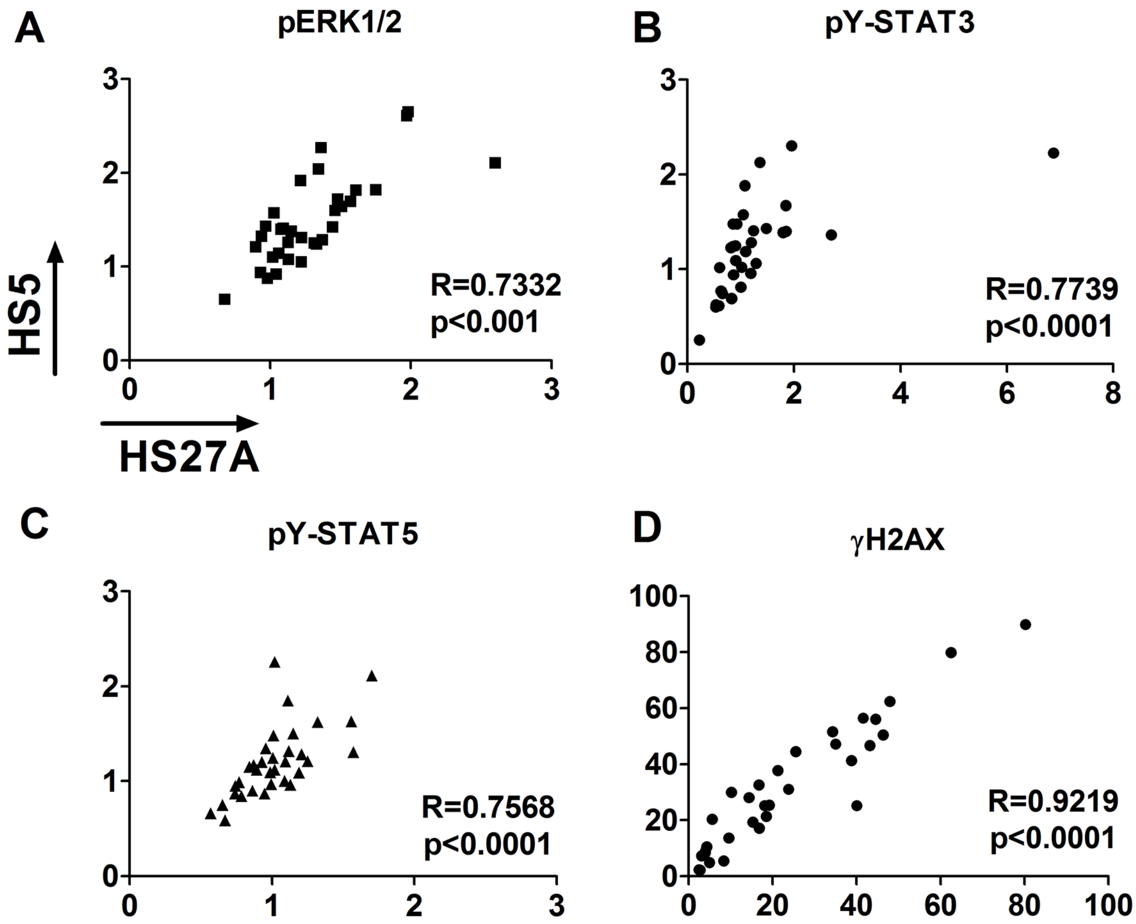

Supplementary Figure 7: HS5 and HS27A stromal cells induce similar signaling responses in AML cells. Pediatric primary AML samples were cultured off or on stroma for 24 hr and pY-STAT3, pY-STAT5 and pERK1/2 were measured by FACS. Bivariate correlation analysis was done between the ΔMFI of pERK1/2, pY-STAT3, pY-STAT5 **(A-C)** induced by HS5 and those induced by HS27A. ΔMFI is defined as in Supplementary Figure 2. Similar correlation is shown for γH2AX induced by 10 μM etoposide, for AML cells on HS5 v HS27A stroma **(D)**. Values shown are the percent γH2AX+ in the live (cPARP-) population. N=34.

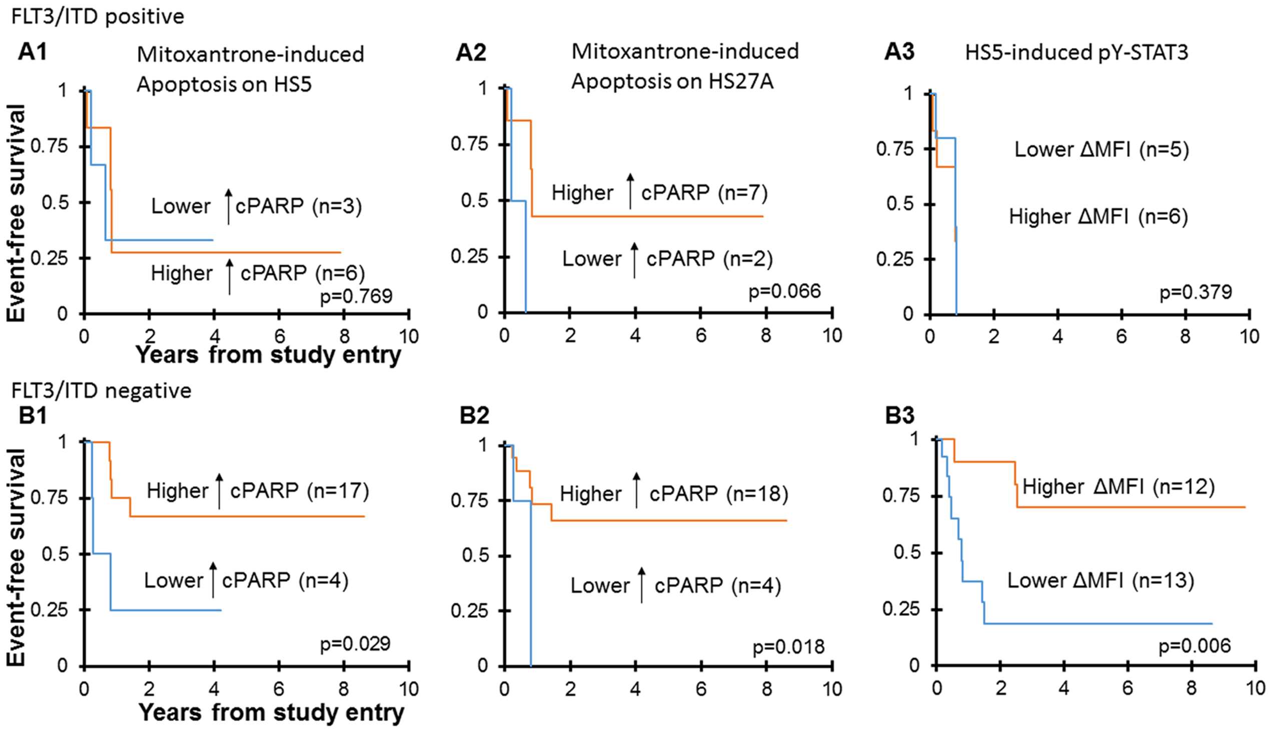

Supplementary Figure 8: FLT3/ITD mutation does not account for the association of poor event-free survival with strong stroma-mediated mitoxantrone resistance and with absent stroma-induced STAT3 activation. Pediatric primary AML samples were cultured off or on stroma, treated with 100 nM mitoxantrone for 24 hours, and apoptosis was quantified by FACS analysis for cPARP **(A1-A2, B1-B2)**. The percentage of spontaneous apoptosis was subtracted from the drug-treated samples to yield the percentage of apoptosis attributed to drug treatment (↑cPARP). Cut point analyses separated the cohort into approximate quartiles. The patients were further divided into two populations based on FLT3/ITD status. For the population with FLT3/ITD mutations, 3-year EFS was not different between patients with different mitoxantrone-induced apoptosis rates on HS5 (A1, n=3 and 6 for the lower and higher group, separately) or HS27A stroma (A2, n=2 and 7 for the lower and higher group, separately). For the population without FLT3/ITD mutations, patients whose AML cells had >3.75% mitoxantrone-induced apoptosis on HS5 stroma had higher 3-year EFS, and those with <3.75% mitoxantrone-induced apoptosis on HS5 stroma had lower 3-year EFS (B1, n=4 and 17 for the lower and higher group, separately). Similarly, patients whose AML cells had >3.43% mitoxantrone-induced apoptosis on HS27A stroma had higher 3-year EFS, and those with <3.43% mitoxantrone-induced apoptosis on HS27A stroma had lower 3-year EFS (B2, n=4 and 18 for the lower and higher group, separately). Patient samples were also cultured on or off HS5 stroma for 24 hours, and pY-STAT3 was measured by FACS **(A3, B3)**. Stroma-induced pY-STAT3 is defined as the fold change in MFI over cells cultured alone (ΔMFI). Cut point analysis separated the cohort in half. Again, the patients were further divided into two populations based on FLT3/ITD status. For the population with FLT3/ITD mutations, 3-year EFS was not different among patients with different HS5-induced pY-STAT3 (A3, n=5 and 6 for the lower and higher group, separately). For the population without FLT3/ITD mutations (B3, n=13 and 12 for the lower and higher group, separately), the patients whose AML cells failed to induce pY-STAT3 (stroma-induced pY-STAT3 < 1.22) when co-cultured with HS5 cells had significantly lower 3-year EFS than those whose AML cells appropriately induced pY-STAT3 (> 1.22). EFS was estimated by the Kaplan-Meier method and groups were compared for significant differences by the log rank test. Patients who underwent stem cell transplant (SCT) were censored for Kaplan-Meier analysis of EFS.

Supplementary Table 1: Clinical characteristics associated with primary pediatric AML patient samples used for this study.

See Supplementary File 1

Supplementary Table 2: Univariable Cox analyses of EFS for each marker in Figure 5 as well as for cytogenetics/mutation risk group

**Univariable Cox analyses**				
	**EFS from study entry**			
	**N**	**HR**	**95% CI**	**p**
**mitoxantrone-induced apoptosis on HS5**				
Higher cPARP	23	1		
Lower cPARP	7	3.20	1.01 - 10.14	**0.049**
**Risk group**				
Standard	5	1		
Low	16	1.34	0.15 - 12.0	0.796
High	7	4.74	0.55 - 40.7	0.156
**mitoxantrone-induced apoptosis on HS27A**				
Higher cPARP	25	1		
Lower cPARP	6	7.41	1.94 - 28.3	**0.003**
**Risk group**				
Standard	6	1		
Low	16	0.72	0.13 - 3.95	0.707
High	7	2.59	0.50 - 13.4	0.256
**HS5-induced pY-STAT3**				
Higher ΔMFI	18	1		
Lower ΔMFI	19	2.82	1.10 - 7.24	**0.032**
**Risk group**				
Standard	10	1		
Low	18	0.84	0.29 - 2.45	0.755
High	6	2.39	0.64 - 8.91	0.193

Notes:
1. EFS: event-free survival
2. Patients who underwent SCT on protocol therapy were censored for analysis of EFS.
3. HR: hazard ratio
4. CI: confidence interval

Supplementary Table 3: Multivariable Cox analyses of EFS for each marker in Figure 5 as well as for cytogenetics/ mutation risk group

**Multivariable Cox analyses**				
	**EFS from study entry**			
	**n**	**HR**	**95% CI**	**p**
**mitoxantrone-induced apoptosis on HS5**				
Higher cPARP	21	1		
Lower cPARP	7	2.8	0.74 - 10.5	0.128
**Risk group**				
Standard	5	1		
Low	16	1.09	0.12 - 10.1	0.939
High	7	3.02	0.32 - 28.8	0.338
**mitoxantrone-induced apoptosis on HS27A**				
Higher cPARP	23	1		
Lower cPARP	6	7.62	1.84 - 31.6	**0.005**
**Risk group**				
Standard	6	1		
Low	16	0.54	0.09 - 3.13	0.495
High	7	2.22	0.42 - 11.7	0.347
**HS5-induced pY-STAT3**				
Higher ΔMFI	17	1		
Lower ΔMFI	17	2.15	0.76 - 6.10	0.149
**Risk group**				
Standard	10	1		
Low	18	0.78	0.27 - 2.27	0.644
High	6	1.59	0.39 - 6.45	0.519

Note:
1. Patients who underwent SCT on protocol therapy were censored for analysis of EFS.
